# CArdiovasculaR Outcomes Based Upon EjectIon Systolic TimE in Patients With ST Elevation Myocardial Infarction (ARISE-STEMI) Study

**DOI:** 10.1016/j.cjco.2024.11.014

**Published:** 2024-11-26

**Authors:** Tyler Szun, Alexander Zaremba, Aleksander Dokollari, Azin Khafipour, Hilary Bews, Seth Cheung, James W. Tam, Shuangbo Liu, Derek So, Sean Van Diepen, Ashish H. Shah

**Affiliations:** a Max Rady College of Medicine, University of Manitoba, Winnipeg, Manitoba, Canada; b Section of Cardiac Surgery, University of Manitoba, Winnipeg, Manitoba, Canada; c Section of Cardiology, Department of Internal Medicine, Max Rady Faculty of Health Sciences, University of Manitoba, Winnipeg, Manitoba, Canada; d University of Manitoba, Winnipeg, MB, Canada; e Institute of Cardiovascular Sciences, St Boniface Hospital, University of Manitoba, Winnipeg, Manitoba, Canada; f University of Ottawa Heart Institute, Ottawa, Canada; g Department of Critical Care Medicine and Division of Cardiology, Department of Medicine, Faculty of Medicine & Dentistry, University of Alberta, Edmonton, Canada

## Abstract

**Background:**

Despite improvements in revascularization, systems of care, and secondary prevention therapies, 30-day mortality rates in patients presenting with ST-elevation myocardial infarction (STEMI) undergoing primary percutaneous coronary intervention (PPCI) remains 4% to 6%. This study aims to investigate the utility of the ejection systolic time (EST) and ejection systolic period (ESP) in identifying high-risk STEMI patients.

**Methods:**

In this retrospective study, consecutive patients with STEMI undergoing PPCI at a tertiary cardiac center between January 2020 and October 2021 were included. EST and ESP were calculated on the MacLab. Univariable and multivariable Cox regression analysis were used to identify risk predictors. The primary outcome was mortality at 30 days.

**Results:**

Six hundred forty-one STEMI patients (mean age: 64.4 ± 13.2 years; 182/641 [28.4%] female patients) were recruited. Within 30 days of presentation, 32 patients (5.0%) died, and they were more frequently older, female, and had higher rates of previous stroke, chronic kidney disease, and dialysis use. Patients dying within 30 days had lower EST (0.20 ± 0.04 vs 0.24 ± 0.04 seconds/beat; *P* < 0.0001) and ESP (17.64 ± 2.66 vs 19.29 ± 2.74 seconds/min; *P* = 0.004). After multivariable modeling, only EST was a significant predictor of early (<30 days) mortality (hazard ratio 4.5, 95% confidence interval 1.7-12.1; *P* = 0.003), prolonged in-hospital stay (>4 days), diuretic use, new diagnosis of heart failure, need for intubation or ventilation, and inotrope and/or vasopressor use during the index hospital admission. ESP and EST were not associated with the mortality between 30 days and 1 year.

**Conclusions:**

A lower EST was associated with mortality at 30 days and in-hospital adverse outcomes. EST may serve as a useful hemodynamic marker to risk-stratify STEMI patients.

## Graphical abstract

**Figure:**
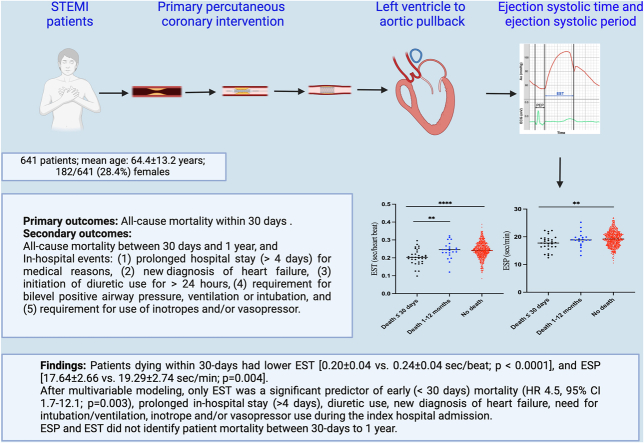

## Résumé

**Contexte:**

Malgré les progrès observés dans les techniques de revascularisation, les systèmes de soins et les traitements de prévention secondaire, le taux de mortalité à 30 jours chez les patients ayant subi un infarctus du myocarde avec élévation du segment ST (STEMI) traités par une intervention coronarienne percutanée primaire (ICPP) est toujours de 4 % à 6 %. Cette étude vise à évaluer l’utilité du temps d’éjection systolique (EST/ejection systolic time) et de la période d’éjection systolique (ESP/ejection systolic period) pour identifier les cas de STEMI à haut risque.

**Méthodologie:**

Cette étude rétrospective a été menée auprès de patients consécutifs ayant subi un STEMI traités par une ICPP dans un établissement de cardiologie tertiaire de janvier 2020 à octobre 2021. L’EST et l’ESP ont été calculés à l’aide du MacLab. Des analyses de régression de Cox univariées et multivariées ont été utilisées pour déterminer les facteurs prédictifs de risque. Le principal critère d’évaluation était la mortalité à 30 jours.

**Résultats:**

Six cent quarante et un patients ayant subi un STEMI (âge moyen : 64,4 ± 13,2 ans; 182/641 [28,4 %] femmes) ont été recrutés. Au cours des 30 jours suivant la consultation, 32 (5,0 %) patients sont décédés; ils étaient généralement plus âgés, de sexe féminin et étaient plus susceptibles d’avoir des antécédents d’AVC, de néphropathie chronique et d’utilisation de dialyse. Les patients qui sont décédés au cours de ces 30 jours affichaient un EST (0,20 ± 0,04 vs 0,24 ± 0,04 seconde/battement; *p* < 0,0001) et une ESP (17,64 ± 2,66 vs 19,29 ± 2,74 secondes/min; *p* = 0,004) plus bas. Après une modélisation multivariée, seul l’EST s’est révélé un facteur prédictif important de mortalité précoce (< 30 jours) (rapport des risques instantanés : 4,5; intervalle de confiance à 95 % : 1,7-12,1; *p* = 0,003); d’hospitalisation prolongée (> 4 jours), d’utilisation de diurétique; de nouveau diagnostic d’insuffisance cardiaque; de besoin d’intubation ou de ventilation et d’utilisation d’inotrope et/ou de vasopresseur pendant l’hospitalisation de référence. L’ESP et l’EST n’ont pas été associés à la mortalité entre 30 jours et 1 an.

**Conclusions:**

Un EST plus bas a été associé à la mortalité à 30 jours et à des issues défavorables pendant l’hospitalisation. L’EST peut servir de marqueur hémodynamique utile pour stratifier le risque chez les patients ayant eu un STEMI.

Primary percutaneous coronary intervention (PPCI) is the gold standard therapy treating patients presenting with ST-elevation myocardial infarction (STEMI).^1^ Despite the success of PPCI, and resultant improved outcomes, these STEMI patients frequently experience adverse outcomes including mortality.^2^ In current day practice, most STEMI patients are discharged within 1-3 days from their presentation; multiple studies have supported expedited discharge post-PPCI as a safer and cost-effective strategy.^3^, ^4^, ^5^ Hence, it is important to differentiate high- vs low-risk STEMI patients, so that appropriate care can be directed at the ones remaining at a high risk for adverse outcomes.

Various risk scoring systems predicting outcomes in acute coronary syndrome (ACS) patients have been described. The original Thrombolysis in Myocardial Infarction (TIMI) risk score was readjusted to predict 1-year outcome in STEMI patients treated with PPCI.^6^ The Global Registry of Acute Cardiac Events (GRACE) risk score estimates mortality at 6 months in patients presenting with ACS.^7^ Similarly, the Zwolle, Primary Angioplasty in Myocardial Infarction (PAMI), and Controlled Abciximab and Device Investigation to Lower Late Angioplasty Complications (CADILLAC) risk scores predict mortality at 1, 6, and 12 months, respectively, among patients investigated by cardiac catheterization.^8^^,^^9^ However, most of the score systems are not used in contemporary practice. Interestingly, intraprocedural hemodynamic parameters collected during the PPCI have been understudied. An ejection systolic period (ESP), a surrogate marker of cardiac output (CO), can be measured during catheter pullback from left ventricle to aorta. Ejection systolic time (EST), a surrogate marker of stroke volume (SV), can be calculated by dividing ESP by heart rate.^10^ We aimed to investigate the utility of intraprocedural EST and ESP in identifying high-risk STEMI patients undergoing PPCI.

## Methods

### Study design and population

**Figure 1:**
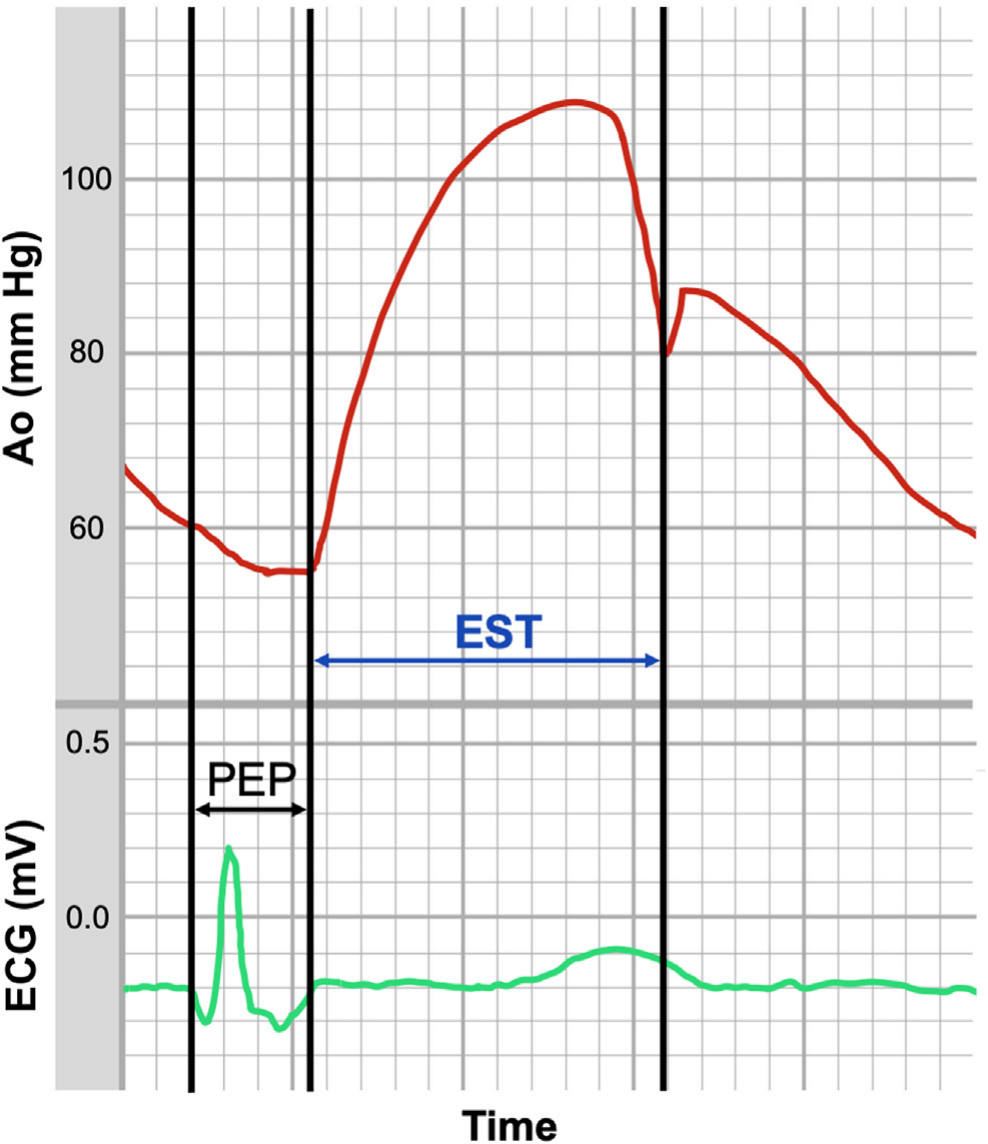
Ejection systolic period calculation. Ao, central aortic pressure; ECG, electrocardiogram; EST, ejection systolic time; PEP, pre-ejection period.

In a retrospective, single-centre study design data from the consecutive STEMI patients undergoing PPCI, at the St Boniface Hospital, Manitoba, Canada during January 2020 to October 2021 were analyzed. The study was approved by the local Research and Ethics Board, and patient consent was waived. The inclusion criteria comprised (1) patients presenting with STEMI, who were (2) treated with PPCI, and had (3) left ventricular (LV) to aortic pullback performed during the procedure. The exclusion criteria were patients with (1) a final diagnosis other than STEMI, (2) referral for coronary artery bypass graft (CABG) surgery, and who had (3) no LV to aortic pull-back or had a poor-quality hemodynamic tracing precluding an EST/ESP measurement. The data collected encompassed demographics, cardiovascular risk factors and comorbidities, and intraprocedural hemodynamic parameters including systolic (SBP) and diastolic blood pressure (DBP), left ventricular end-diastolic pressure (LVEDP), and calculated coronary perfusion pressure (DBP-LVEDP; mm Hg). While performing LV to aortic pullback, the MacLab (GE Health Care, Boston, MA) system allows calculating ESP (in seconds/min); dividing ESP by the heart rate provided EST (in seconds/beat), as described in Figure 1. STEMI was defined according to the American Heart Association Guidelines.

### Outcomes

All outcome data were obtained through electronic medical records. The primary outcome was all-cause mortality within 30 days from presentation. The secondary outcomes included all-cause mortality between 30 days and 1 year of presentation with STEMI, and in-hospital events including prolonged hospital stay (>4 days) for medical reasons (as a result of heart failure, or arrhythmia), new diagnosis of heart failure (as defined by the American Heart Association guidelines),^11^ initiation of diuretic use for >24 hours, requirement for bilevel positive airway pressure (BiPAP), ventilation or intubation, and requirement for use of inotropes and/or vasopressors.

### Statistical analysis

Baseline characteristics and angiographic information were expressed either as mean ± standard deviation or median with interquartile range. Categorical data were presented as numbers and percentages. Continuous variable differences were assessed using either Tukey multiple comparisons tests, Student *t* test, or Wilcoxon rank-sum test, whereas categorical variables were analyzed with the χ^2^ test or Fisher exact test. We performed a multivariable Cox regression with all preoperative characteristics (age, sex, height, weight, and body mass index, cardiovascular risk factors and comorbidities, and intraprocedural hemodynamics including systolic, diastolic, and LVEDP). The assumptions of proportionality were checked, and age was included as a time-dependent covariate. Finally, we built a parsimonious model by including all preinterventional variables and running a forward selection with a high *P* value inclusion (*P* < 0.6). All variables that met the criteria were then entered into another forward selection with Akaike information criterion (AIC) in the order of the first forward selection. The lowest AIC model was determined to be the best fit for the data. We then checked the area under the curve and ran a k-fold cross-validation with 10 folds and bootstrap 95% confidence intervals (CIs) to calibrate and validate the model.

EST and ESP were divided into 2 groups using the median values (<0.24 and ≥0.24 and <19.2 and ≥19.2, respectively). We compared death outcomes between the EST and ESP groups using a Cox regression analysis, and the other outcomes were compared with a logistic regression. The model was adjusted for age, female, body mass index, hypertension, diabetes mellitus, dyslipidemia, prior stroke, peripheral vascular disease (PVD), chronic kidney disease (CKD), dialysis, ischemic heart disease, PCI, or CABG, LVEDP, and ESP or EST depending on the comparison. We checked for multicollinearity with a variance inflation factor and decided to exclude systolic BP, diastolic BP, heart rate, and DBP-LVEDP as covariates because of collinearity. Result are reported as adjusted hazard ratio and survival Kaplan Meier graphs for the EST and ESP groups for <30-day and >30-day mortality.

## Results

### Baseline demographics

During the study period, 800 individual patients were brought to the catheter laboratory at the St Boniface Hospital with an initial diagnosis of STEMI. A total of 159 patients met exclusion criteria, and the final study included 641 patients (Supplemental Fig. 1). STEMI patients were on average 64.4 ± 13.2 years of age (32-101 years); 182 (28.4%) were female and had a mean body mass index of 28.9 ± 5.5. Cardiovascular risk factors were prevalent within the population, including hypertension (57.9%), diabetes (25.7%), dyslipidemia (40.7%), prior stroke or transient ischemic attack (TIA) (4.8%), PVD (3.0%), CKD (6.1%), and regular dialysis treatment (0.9%). A history of ischemic heart disease was present in 19.0% of the population, whereas 14.5% had a history of previous PCI or CABG. Baseline patient demographics are presented in Table 1.

**Table 1:** Demographics, risk factors, and cardiac catheterization data by primary outcome subgroups

	Study population (n=641)	Outcome subgroups				
		No death (n=586)	Death <30 d (n=32)	*P* value	30 d < death < 1 y (n=23)	*P* value
Demographics						
Age, y	64.4 ± 13.2	63.7 ± 13.0	73.5 ± 12.9	0.0001	68.4 ± 12.8	0.21
Female	182 (28.4)	158 (27.0)	15 (46.9)	0.02	9 (39.1)	0.20
Height, cm	171.7 ± 10.1	172.0 ± 10.0	168.9 ± 11.2	0.2	169.2 ± 11.5	0.38
Weight, kg	85.4 ± 19.4	86.0 ± 18.9	79.8 ± 17.9	0.18	79.0 ± 17.7	0.19
BMI	28.9 ± 5.5	29.0 ± 5.5	27.9 ± 4.7	0.49	26.8 ± 3.9	0.13
Risk factors						
Hypertension	371 (57.88)	333 (56.88)	21 (65.63)	0.32	17 (73.91)	n/a
Diabetes mellitus	165 (25.74)	146 (24.96)	8 (25.00)	0.99	11 (47.83)	n/a
Dyslipidemia	261 (40.72)	243 (41.26)	9 (28.13)	0.13	9 (39.13)	n/a
Prior stroke (TIA)	31 (4.84)	23 (3.74)	5 (15.63)	0.003	3 (13.04)	0.004
PVD	19 (2.96)	15 (2.55)	1 (3.13)	0.84	3 (13.04)	0.004
CKD	39 (6.08)	24 (4.07)	6 (18.75)	0.0002	9 (39.13)	<0.0001
Dialysis	6 (0.94)	1 (0.17)	2 (6.25)	<0.0001	3 (13.04)	<0.0001
Ischemic heart disease	122 (19.03)	113 (19.19)	6 (18.75)	0.94	3 (13.04)	n/a
PCI or CABG	93 (14.51)	88 (14.94)	3 (9.38)	0.38	2 (8.70)	n/a
Hemodynamic data						
SBP, mm Hg	117.7 ± 24.1	119.0 ± 21.3	93.5 ± 21.9	<0.0001	116.5 ± 12.3	0.86
DBP, mm Hg	69.0 ± 14.2	69.9 ± 13.5	57.8 ± 11.3	<0.0001	62.0 ± 8.4	0.02
LVEDP, mm Hg	22.8 ± 8.7	22.7 ± 7.8	25.8 ± 7.0	0.10	20.7 ± 5.4	0.48
DBP-LVEDP, mm Hg	46.6 ± 17.4	47.5 ± 13.3	32.9 ± 8.0	<0.0001	41.1 ± 9.0	0.07
ESP, s/min	19.20 ± 3.30	19.29 ± 2.74	17.64 ± 2.66	0.004	18.95 ± 2.70	0.84
Heart rate, bpm	80.8 ± 16.2	80.5 ± 15.3	88.2 ± 13.3	0.01	78.5 ± 11.0	0.82
EST, s/beat	0.24 ± 0.69	0.24 ± 0.04	0.20 ± 0.04	<0.0001	0.25 ± 0.03	0.96

Data presented as n (%) or mean ± standard deviation.

BMI, body mass index; bpm, heartbeats per minute; CABG, coronary artery bypass graft; CKD, chronic kidney disease; DBP, diastolic blood pressure; ESP, ejection systolic period; EST, ejection systolic time; LVEDP, left ventricular end-diastolic pressure; n/a, not available; PCI, percutaneous coronary intervention; PVD, peripheral vascular disease; SBP, systolic blood pressure; TIA, transient ischemic attack.

### Catheterization and hemodynamic parameters

Their mean SBP was 117.7 ± 24.1 mm Hg, the mean DBP was 69.0 ± 14.2 mm Hg, the mean heart rate was 80.8 ± 16.2 beats/min, and the mean LVEDP was 22.8 ± 8.7 mm Hg. The mean ESP was 19.20 ± 3.30 seconds/min (range 7.3-35.2 seconds/min), and the mean EST was 0.24 ± 0.69 seconds/beat (range 0.08-0.39 seconds/beat). Hemodynamic data for the entire STEMI population is summarized in Table 1.

### Outcome data

A total of 32 patients (5.0%) met the primary outcome of death within 30 days of hospital presentation. Secondary outcomes occurred in STEMI patients as follows: 23 (3.6%) additional patients died between 30 days and 1 year, 93 (14.5%) were hospitalized for more than 4 days because of a medical condition, 73 (11.4%) received a new diagnosis of heart failure, 63 (9.8%) required new initiation of diuretics for more than 24 hours, 40 (6.2%) required BiPAP or invasive ventilation, and 75 (11.7%) required hemodynamic support with inotropes and/or vasopressors use.

### Association between demographics and cardiovascular risk profile on the outcomes

Patients who died within 30 days were older (73.5 ± 12.9 vs 63.7 ± 13.0 years; *P* = 0.001) and were likely to have a history of prior stroke or TIA (15.6% vs 3.7%, *P* = 0.003), CKD (18.8% vs 4.1%, *P* = 0.0002), and dialysis use (6.2% vs 0.17%, *P* < 0.0001). In our comparison of the patients dying between 30 days and a year vs those alive, age was not a significant parameter; however, they were likely to have a history of prior stroke or TIA (13.0% vs 3.7%, *P* = 0.004), PVD (13.0% vs 2.6%, *P* = 0.004), CKD (39.1% vs 4.1%, *P* < 0.0001), and dialysis use (13.0% vs 0.2%, *P* < 0.0001). Although female patients constituted only 28.4% of the total study cohort, they represented a larger proportion (15 of 32; 46.9%) of the patients who died within 30 days (Table 1).

### Hemodynamic parameters and their relationship to the outcomes

The STEMI patients who died within 30 days had a significantly lower mean SBP (93.5 ± 21.9 vs 119.0 ± 21.3 mm Hg, *P* < 0.0001), DBP (57.8 ± 11.3 vs 69.9 ± 13.5 mm Hg, *P* < 0.0001), and DBP-LVEDP, a surrogate for coronary perfusion pressure (32.9 ± 8.0 mm Hg vs 47.5 ± 13.3 mm Hg, *P* < 0.0001), but a higher heart rate (88.2 ± 13.3 vs 80.5 ± 15.3 beats/min, *P* = 0.0185). On the other hand, low DBP was the only hemodynamic variable significantly different between patients who died between 30 days and 1 year, and those alive at 1 year (62.0 ± 8.4 vs 69.9 ± 13.5 mm Hg, *P* = 0.0208) (Table 1).

### EST-ESP and outcomes

**Figure 2:**
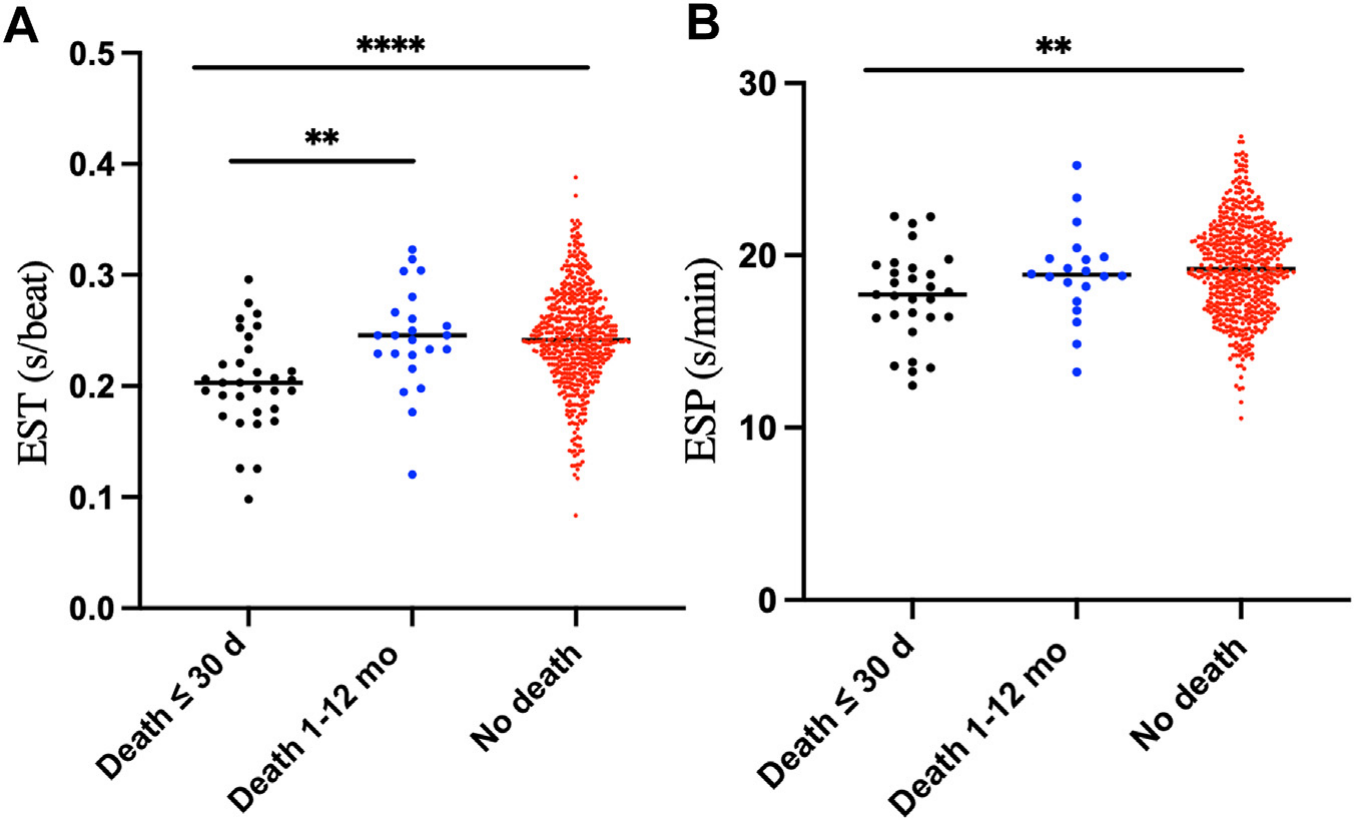
Association of EST and ESP with death. Mean (**A**) EST and (**B**) ESP of the study population, grouped by death within 30 days, death (30 days to 1 year), and no death within 1 year. ESP, ejection systolic period; EST, ejection systolic time. ∗*P* < 0.05, ∗∗*P* < 0.01, ∗∗∗*P* < 0.01, ∗∗∗∗*P* < 0.0001.

**Figure 3:**
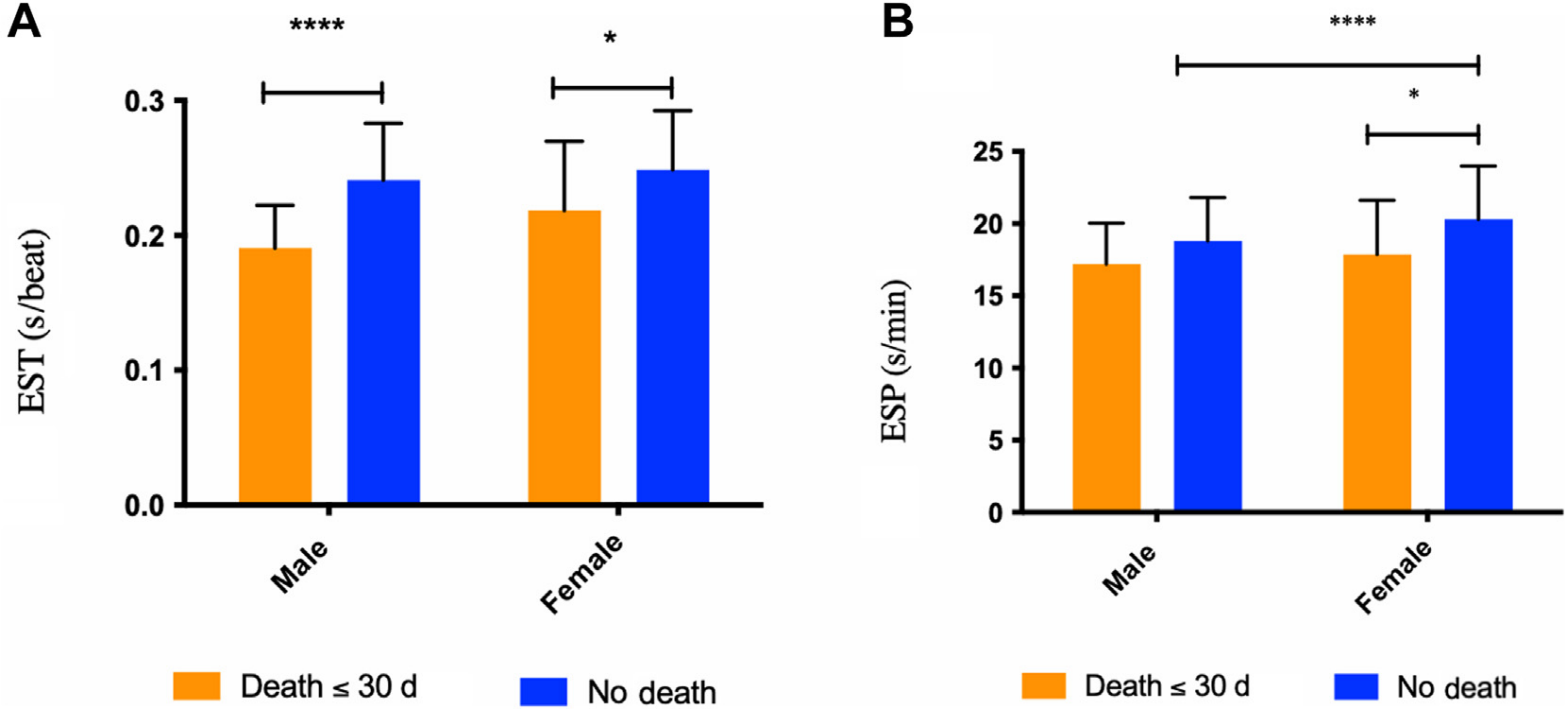
Association of EST and ESP with death within 30 days and the impact of biological sex. Mean (**A**) EST and (**B**) ESP for male and female patients comparing death within 30 days after presentation with STEMI vs not. ESP, ejection systolic period; EST, ejection systolic time; STEMI, ST-elevation myocardial infarction. ∗*P* < 0.05, ∗∗*P* < 0.01, ∗∗∗*P* < 0.01, ∗∗∗∗*P* < 0.0001.

Patients dying within 30 days from their presentation with STEMI were noted to have significantly lower EST (0.20 ± 0.04 vs 0.24 ± 0.04 seconds/min; *P* < 0.0001) and ESP (17.64 ± 2.66 vs 19.29 ± 2.74 seconds/min; *P* = 0.004). No difference was observed between the patients dying between 30 days and 1 year vs those alive (Fig. 2, A and B). Female patients dying within 30 days demonstrated higher EST than their male counterparts experiencing mortality within the same period. Despite observing higher mortality among elderly and female patients, ESP and EST were similar for patients irrespective of their age or biological sex (Fig. 3, A and B).

As the patients dying within 30 days from their presentation were noted to be older and having lower EST and ESP, we assessed the relationship between age and biological sex with EST and ESP. Age and biological sex did not affect EST or ESP among all-comer patients (Supplemental Fig. 2).

The median EST was 0.24 seconds/beat, and hence we used it as a cutoff point to evaluate the impact of EST on the outcomes. Low EST was significantly associated with mortality within 30 days (HR 4.5, 95% CI 1.7-12.1; *P* = 0.003). Although no such relationship between EST and death between 30 days and 1 year was identified, low EST was strongly associated with all other secondary outcomes. Unadjusted and multivariable adjusted modeling-based analysis for EST is described in Table 2.

**Table 2:** The relationship of ejection systolic time to the outcomes

	Unadjusted model			Multivariable adjusted model		
	EST < 0.24 HR (95% CI)	EST ≥ 0.24 HR (95% CI)	*P* value	EST < 0.24 HR (95% CI)	EST ≥ 0.24 HR (95% CI)	*P* value
Death <30 d	4.4 (1.8, 10.7)	0.2 (0.1, 0.6)	0.001	4.5 (1.7, 12.1)	0.2 (0.1, 0.6)	0.003
Death: 30 d to 1 y∗	1.3 (0.5, 3.2)	0.8 (0.3, 1.9)	0.549	1.3 (0.5, 3.3)	0.8 (0.3, 2.0)	0.616
During the index hospital admission						
Prolonged in-hospital stay (>4 d)	2.8 (1.7, 4.6)	0.4 (0.2, 0.6)	<0.0001	2.6 (1.5, 4.6)	0.4 (0.2, 0.7)	0.001
New diagnosis of HF	2.7 (1.5, 5.0)	0.4 (0.2, 0.7)	0.001	2.4 (1.3, 4.7)	0.4 (0.2, 0.8)	0.008
Diuretic use >24 h	4.4 (2.3, 8.6)	0.2 (0.1, 0.4)	<0.0001	4.5 (2.1, 9.3)	0.2 (0.1, 0.5)	<0.0001
Intubation, ventilation, BiPAP	3.9 (1.8, 8.5)	0.3 (0.1, 0.5)	<0.0001	4.4 (1.8, 10.7)	0.2 (0.1, 0.6)	0.001
Inotropes or vasopressors use	2.2 (1.3, 3.7)	0.5 (0.3, 0.8)	0.002	2.1 (1.2, 3.7)	0.5 (0.3, 0.8)	0.009

Adjusted for age, gender, body mass index, hypertension, diabetes mellitus, dyslipidemia, prior stroke, or transient ischemic attack, peripheral vascular disease, chronic kidney disease, dialysis, ischemic heart disease, percutaneous coronary intervention or coronary artery bypass graft, left ventricular end-diastolic pressure, and ejection systolic period.

BiPAP, bilevel positive airway pressure; CI, confidence interval; EST, ejection systolic time; HF, heart failure; HR, hazard ratio.

∗ Those who died before 30 days were excluded.

An average ESP was noted to be low among patients dying within 30 days from their presentation. Using the median value of 19.2 seconds/min to create 2 groups, unadjusted modeling demonstrated that low ESP was associated with new-onset heart failure, diuretic use for >24 hours, and need for inotropes and/or vasopressor therapies. However, post multivariable modeling, no such relationship persisted (Table 3).

**Table 3:** The relationship of the ejection systolic period to secondary outcomes in the study

	Unadjusted model			Multivariable adjusted model		
	ESP < 19.2,∗ HR (95% CI)	ESP ≥ 19.2 HR (95% CI)	*P* value	ESP < 19.2,∗ HR (95% CI)	ESP ≥ 19.2 HR (95% CI)	*P* value
Death <30 d use	2.1 (0.9, 4.6)	0.4 (0.2, 1.0)	0.063	1.5 (0.6, 3.7)	0.7 (0.3, 1.6)	0.372
30 d <death <1 y use	1.4 (0.5, 3.4)	0.7 (0.3, 1.8)	0.507	1.6 (0.6, 4.0)	0.6 (0.2, 1.6)	0.341
During the index hospital admissionuse						
Prolonged stay in hospital (>4 d) use	1.4 (0.9, 2.2)	0.7 (0.4, 1.2)	0.192	1.5 (0.9, 2.5)	0.7 (0.4, 1.1)	0.124
New diagnosis of HF use	2.1 (1.2, 3.8)	0.5 (0.3, 0.8)	0.012	1.7 (0.9, 3.4)	0.6 (0.3, 1.1)	0.104
Diuretic use >24 h use	2.1 (1.1, 3.7)	0.5 (0.3, 0.9)	0.016	1.8 (0.9, 3.4)	0.6 (0.3, 1.1)	0.094
Intubation, ventilation, BiPAP use	1.5 (0.8, 3.0)	0.6 (0.3, 1.3)	0.199	1.2 (0.5, 2.7)	0.8 (0.4, 1.9)	0.674
Inotropes/vasopressors use	1.8 (1.1, 2.9)	0.6 (0.3, 0.9)	0.029	1.6 (0.9, 2.8)	0.6 (0.4, 1.1)	0.116

Adjusted for age, gender, body mass index, hypertension, diabetes mellitus, dyslipidemia, prior stroke, or transient ischemic attack, peripheral vascular disease, chronic kidney disease, dialysis, ischemic heart disease, percutaneous coronary intervention or coronary artery bypass graft, left ventricular end-diastolic pressure, and ejection systolic time.

BiPAP, bilevel positive airway pressure; CI, confidence interval; ESP, ejection systolic period; HF, heart failure; HR, hazard ratio.

∗ Those who died before 30 days were excluded.

**Figure 4:**
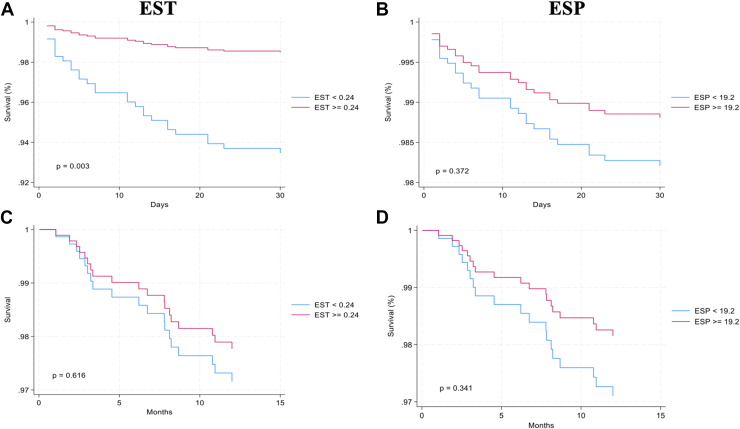
Hazard ratio graphs for death at 30 days from STEMI presentation. Hazard ratio graph describing survival at 30 days and between 30 days and 1 year using EST (**A** and **C**, respectively), and ESP (**B** and **D**, respectively). ESP, ejection systolic period; EST, ejection systolic time; STEMI, ST-elevation myocardial infarction.

To further explore the relationship between EST, ESP, and mortality among STEMI patients, we created a survival function (Fig. 4) and Kaplan-Meier graphs (Supplemental Fig. 3). Only EST, but not ESP, was noted to have significant impact on the survival function up to 30 days (Fig. 4, A and B, respectively). Neither EST nor ESP had any significant association with mortality between 30 days and 1 year (Fig. 4, C and D, respectively). The graphical abstract depicts the study flow, outcomes, and the findings (Central Figure).

## Discussion

In the present study, we demonstrate the prognostic utility of EST in identifying high-risk STEMI patients treated with PPCI during the index hospital admission and at a short-term follow-up. Our observed mortality at 30 days and 1 year among STEMI patients were similar to that in other studies.^1^^,^^12^

Although age is a well-known risk factor for mortality in the setting of myocardial infarction (MI),^13^ our analysis did not show an association between ESP or EST and age. This finding further supports the independent predictive value of ESP and EST.^14^ Furthermore, we described sex-specific mortality differences; although less than one-third of the STEMI cohort were female, they made up nearly 50% of the mortality at 30 days. This sex-related outcome difference has been previously described.^15^^,^^16^ There are a variety of plausible explanations describing such a discrepancy, including that women may present with atypical symptoms more commonly and they are less likely to receive PCI than men. However, our patients did present in a timely fashion, and each were treated with PPCI. Interestingly, female patients dying within 30 days demonstrated higher EST than their male counterparts. Similarly, other studies have also described that despite excess mortality, female patients were more likely to have preserved LV function.^17^ Others have described that excess mortality among female patients is specifically limited to younger patients (<60 years).^15^ Hence, the precise pathophysiology leading to this observed mortality difference remains unclear and cannot be described based on hemodynamic parameters alone. Observed cardiovascular risk factors associated with excess mortality (PVD, stroke/TIA, CKD, or dialysis) in our cohort have also been previously linked with mortality in STEMI patients undergoing PPCI.^18^, ^19^, ^20^

EST is a surrogate marker of stroke volume and has previously demonstrated its predictive capacity in patients with primary pulmonary hypertension,^21^ ischemic heart disease,^22^ and incident heart failure (HF).^23^ Patel et al.^24^ evaluated the association between EST by echocardiography and 1-year outcomes in 545 patients with HF. They demonstrated that the median EST was shorter in patients with HF with reduced ejection fraction (HFrEF) (280 milliseconds [ms]) compared to patients with heart failure with preserved ejection fraction (HFpEF; 315 ms).^24^ Furthermore, higher EST was associated with a lower odds of death or HF hospitalization in patients with HFrEF, but not HFpEF.^24^ Alhakak et al. described a population of 997 HFrEF patients who underwent echocardiographic determination of EST. At a median follow-up of 3.4 years, they found that mortality increased by 9% for every 10-ms decrease in EST, which remained significant even after multivariate adjustment.^25^

To our knowledge, the present study is the first contemporary cohort in which the invasive measurement of EST was evaluated as a prognostic marker in STEMI patients. Patients with myocardial infarction were noted to have low stroke volume but preserved cardiac output due to compensatory increase in heart rate.^26^^,^^27^ Our findings also describe low EST observed in patients with adverse outcomes; however, because their heart rate was higher, it is plausible that compensated ESP (EST × HR) may have eliminated its prognostic value. Other studies were conducted in ACS cohorts prior to the routine use of PPCI or used noninvasive measurement techniques. Haiden et al.^28^ evaluated 852 patients undergoing cardiac catheterization for suspected coronary artery disease (CAD), with 70% of the cohort having significant CAD. Using noninvasive radial tonometry, they calculated the left ventricular ejection time index and identified a relationship with age, SBP, DBP, pulse pressure, and N-terminal pro–brain natriuretic peptide. Furthermore, they observed increased mortality in patients with both the shortest and longest ejection times, with mortality following a U-shaped distribution. Northover et al.^22^ used simultaneous electrocardiogram, phonocardiogram, and carotid pulse tracings to calculate the pre-ejection period (PEP), which included electromechanical delay and isovolumic contraction time, to ejection period (EP or EST) ratio in patients with acute MI within 24 hours of hospitalization. In-hospital mortality was significantly associated with PEP/EP; mortality was 4% in patients with a PEP/EP <0.3, compared with 60% in those with a PEP/EP >0.4.^22^ A study conducted in United Kingdom, where PEP/EP was measured in patients with acute MI who survived the first 7 days, demonstrated that the association between PEP/EP and survival persisted at 1 year.^29^

The prognostic value of EST likely stems from its role as a surrogate marker for stroke volume, the most fundamental hemodynamic parameter.^10^ Logically, patients with myocardial impairment having a low EST would be at a higher risk of short-term mortality. However, after 30 days post-MI, increased proportion of death may be arrhythmogenic or non-cardiac, for which hemodynamic parameters may be nonpredictive. A study of 2804 STEMI patients followed for a median of 4.7 years to characterize temporal etiologies of death. At 30 days, the all-cause mortality rate was 7.9%, mainly attributed to cardiogenic shock or anoxic brain injury following cardiac arrest. However, among patients who survived the initial 30 days, the annual mortality rate was less than 1.5%, with 65% of deaths being noncardiac at 1 year.^30^

In addition to EST, DBP-LVEDP, which serves as a surrogate for coronary perfusion pressure, was significantly lower in patients with early (<30-day) mortality. Buchanan et al. measured coronary perfusion pressure in 922 patients with ACS, of whom 25% had STEMI.^31^ Although perfusion pressure was significantly lower in patients with STEMI compared to those with non-STEMI, the authors did not find this parameter to be predictive of the composite outcome, including in-hospital mortality, MI, HF, and cardiogenic shock.^31^ Additional hemodynamic parameters available at the time of index catheterization have been evaluated in previous studies. Sola et al.^32^ measured the SBP/LVEDP ratio in 219 patients with STEMI. In this study, an SBP/LVEDP ≤4 was associated with an increased risk of in-hospital death (32% vs 5.3%, *P* < 0.0001) or intra-aortic balloon pump (IABP) use (51.6% vs 9.6%, *P* < 0.0001).^32^ Elevated LVEDP alone is well known to be associated with increased risk in STEMI patients.^33^ Interestingly, use of hemodynamic support devices like the Impella (Abiomed, Danvers, MA) in high-risk CAD is associated with improved coronary perfusion.^34^

### Limitations

This is a single-center study, and future studies could seek to externally validate these results. The hemodynamic parameters were measured at one time point. It is plausible that if measured pre- and post-PPCI, these values may change. There are many score systems created to identify high-risk STEMI patients. Although our study did not use any existing scores to compare them with EST, we are working on our next project comparing EST to the known conventional risk scores not only as an independent predictor but also as an additive parameter with an intention to develop a better predictive score. Additionally, we have not collected TIMI flow rate or myocardial blush grade. Although, such parameters may provide added value, and it is likely that patients with compromised TIMI or myocardial blush would have low EST. Mortality was documented by reviewing the electronic patient records more than 2 years after the study recruitment was completed. Although the electronic patient record updates mortality data across the province, some of the deaths may not have been documented in a timely fashion.

## Conclusions

In a large retrospective contemporary cohort of patients with STEMI undergoing PPCI, our exploratory study observed that EST is associated with 30-day mortality. These data suggest that intraprocedural EST may offer a valuable hemodynamic parameter to help risk-stratify patients with STEMI. Such data should be validated in a larger prospective study.
