# Supplementary material for: CArdiovasculaR Outcomes Based Upon EjectIon Systolic TimE in Patients With ST Elevation Myocardial Infarction (ARISE-STEMI) Study
Source: CJC Open. 2024 Nov 26;7(4):516–24. doi: 10.1016/j.cjco.2024.11.014 (PMC12105750; doi:10.1016/j.cjco.2024.11.014)

Supplementary Figure 1: Flow diagram of the study.

Supplementary Figure 2: Age and biological-sex relationship to EST and ESP.

EST (A) and ESP (B) of the study population by age. Males shown in blue, females shown in orange.

Supplementary Figure 3: Kaplan-Meier survival graph and the impact of EST and ESP.

Kaplan-Meier survival graph describing survival at 30-days and between 30-days to 1 year using EST (A and C respectively), and ESP (B and D respectively).

Supplementary Figure 1:

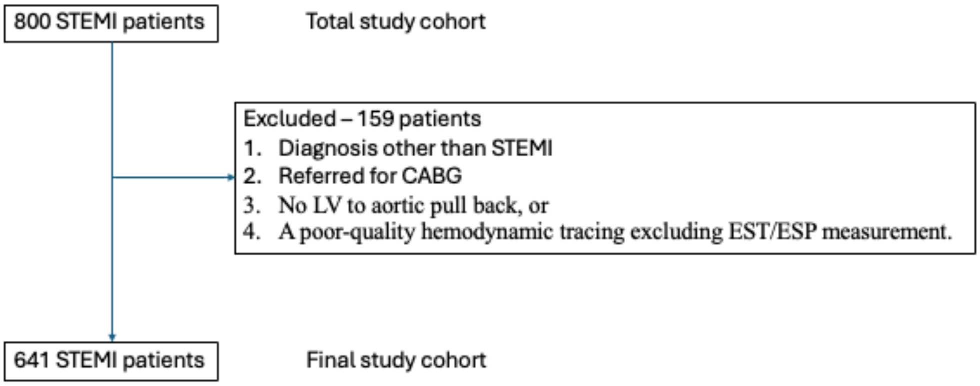

Supplementary Figure 2:

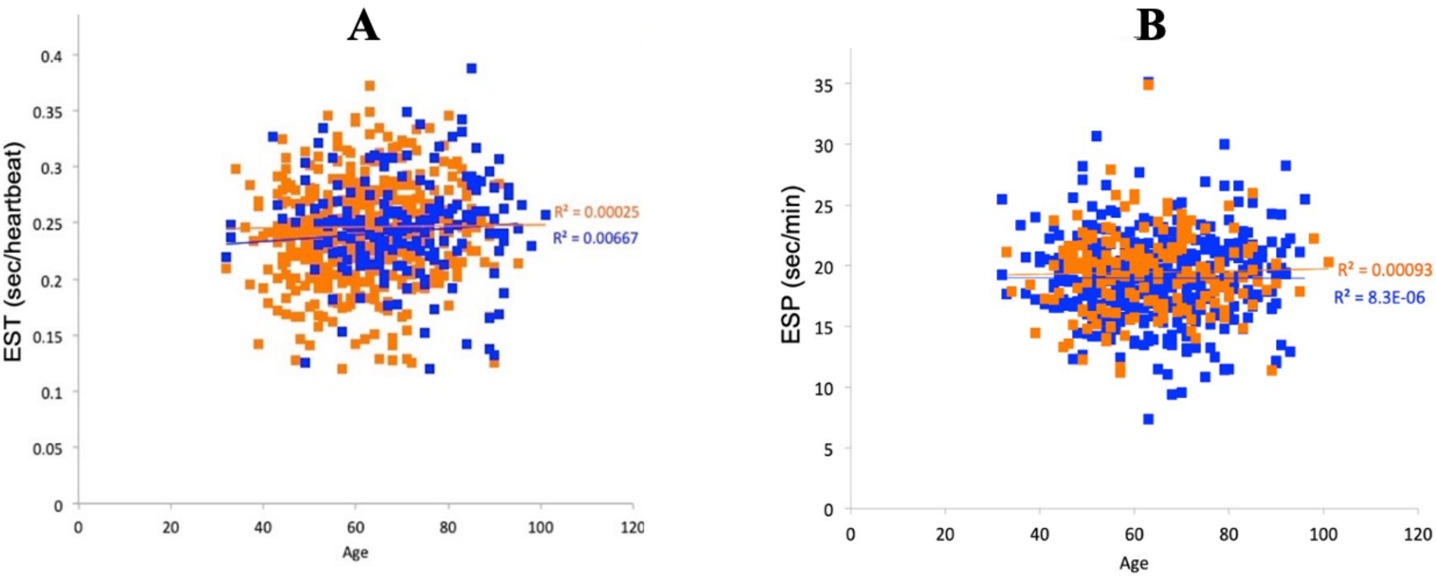

Supplementary Figure 3:

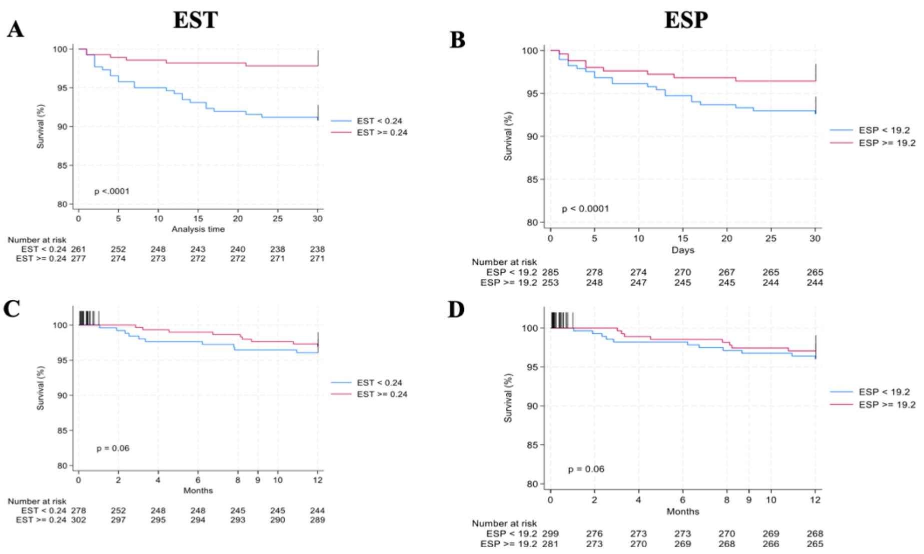

Supplement: Supplementary figures [file mmc1.pdf]
